# Influence of anti-osteoporosis treatments on the incidence of COVID-19 in patients with non-inflammatory rheumatic conditions

**DOI:** 10.18632/aging.104117

**Published:** 2020-10-20

**Authors:** Josep Blanch-Rubió, Natalia Soldevila-Domenech, Laura Tío, Jone Llorente-Onaindia, Manuel Ciria-Recasens, Luciano Polino, Alba Gurt, Rafael de la Torre, Rafael Maldonado, Jordi Monfort, the Covidmar Study Group

**Affiliations:** 1 Rheumatology Service, Hospital del Mar, Passeig Marítim, Barcelona 08003 , Spain; 2 IMIM (Hospital del Mar Medical Research Institute), PRBB, Barcelona 08003, Spain; 3 Integrative Pharmacology and Systems Neuroscience Research Group, Neurosciences Research Program, IMIM-Institut Hospital del Mar d’Investigacions Mèdiques, PRBB, Barcelona 08003, Spain; 4 Laboratory of Neuropharmacology, Department of Experimental and Health Sciences, Universitat Pompeu Fabra, PRBB, Barcelona 08003, Spain; 5 CAP Vila Olímpica, Parc Sanitari Pere Virgili, Barcelona 08005, Spain; 6 Spanish Biomedical Research Centre in Physiopathology of Obesity and Nutrition (CIBERObn), Instituto de Salud Carlos III (ISCIII), Madrid 28029, Spain; 7 Department of Experimental and Health Sciences, Universitat Pompeu Fabra, PRBB, Barcelona 08003, Spain

**Keywords:** denosumab, zoledronate, calcium, vitamin D, anti-resorptive drugs, COVID-19

## Abstract

Coronavirus disease 19 (COVID-19) is currently a global pandemic that affects patients with other pathologies. Here, we investigated the influence of treatments for osteoporosis and other non-inflammatory rheumatic conditions, such as osteoarthritis and fibromyalgia, on COVID-19 incidence. To this end, we conducted a cross-sectional study of 2,102 patients being treated at the Rheumatology Service of Hospital del Mar (Barcelona, Spain). In our cohort, COVID-19 cumulative incidence from March 1 to May 3, 2020 was compared to population estimates for the same city. We used Poisson regression models to determine the adjusted relative risk ratios for COVID-19 associated with different treatments and comorbidities. Denosumab, zoledronate and calcium were negatively associated with COVID-19 incidence. Some analgesics, particularly pregabalin and most of the studied antidepressants, were positively associated with COVID-19 incidence, whereas duloxetine presented a negative association. Oral bisphosphonates, vitamin D, thiazide diuretics, anti-hypertensive drugs and chronic non-steroidal anti-inflammatory drugs had no effect on COVID-19 incidence in the studied population. Our results provide novel evidence to support the maintenance of the main anti-osteoporosis treatments in COVID-19 patients, which may be of particular relevance to elderly patients affected by the SARS-CoV-2 pandemic.

## INTRODUCTION

Infections by SARS-CoV-2, a novel coronavirus that emerged in China in late 2019 [1], and the disease that it causes, COVID-19, became a global pandemic on March 11^th^, 2020 [2]. By July19^th^, 2020, COVID-19 had infected 14,348,475 people and caused 603,167 deaths worldwide [3]. The incidence of COVID-19 is heterogeneous within different countries. In Spain, the area of Barcelona (Catalonia, Spain) has suffered one of the highest rates of incidence and deaths in Europe, mostly between March and April of 2020 [4].

COVID-19 initially has a viral phase with symptoms that include fever, dry cough, anosmia/ageusia, odynophagia and diarrhea, among others. Approximately seven days after this initial phase, some patients develop a systemic pro-inflammatory state and progress to more severe symptoms, such as dyspnea, shortness of breath, pulmonary infiltrates and hypoxemia. The progression of the disease has been associated with a hyper inflammatory response with high levels of inflammatory markers and pro-inflammatory cytokines, sometimes accompanying a pro-coagulation state. Patients following this evolution sometimes become seriously ill, often requiring admission to Intensive Care Units, and some ultimately may die [5].

The innate and acquired immune responses play a crucial role in the progression of the disease. The immune system seems to be dysregulated in severe forms of COVID-19, probably due to abnormal responses by monocytes, macrophage, and/or dendritic cells [6]. Some disease-modifying, anti-rheumatic drugs used in the treatment of immune-mediated inflammatory diseases may have a protective effect, such as the IL6 antagonists tocilizumab and sarilumab [7], which are currently off-label used to treat patients. Our team (article under revision) and other researchers [8–10] have also shown that some of these anti-rheumatic treatments reduce COVID-19 incidence.

Among the diseases treated by rheumatology, osteoporosis is an age-related chronic disease that affects tens of millions people worldwide, requiring long-term treatment [11]. It is a global chronic pandemic causing enormous morbidity, mortality and economic burdens [12]. The possible effects of anti-osteoporosis pharmacologic treatments in the clinical expression and incidence of COVID- 19 remain unknown. Nonetheless, the most prominent organizations for rheumatology and bone studies, such as the American College of Rheumatology (ACR) [13], the European League Against Rheumatism (EULAR) [14], the American Society for Bone and Mineral Research (ASBMR) [15], and the International Osteoporosis Foundation (IOF) [16], currently do not recommend discontinuing the administration of rheumatologic drugs to treat COVID-19 due to their likely neutral effects. However, such recommendations are based on expert opinions and, to our knowledge, no data are available regarding the safety of using such drugs to treat COVID-19.

Determining whether anti-osteoporosis treatments are safe for COVID-19 patients and whether they influence COVID-19 incidence and its clinical expression could positively impact patient prognosis. These questions apply to all anti-osteoporosis compounds including denosumab, a fully human monoclonal antibody against RANK-L, that inhibits osteoclastogenesis. Denosumab is widely used to treat osteoporosis, but also to prevent skeletal-related events in advanced malignancies with bone metastasis [17]. The RANKL/RANK system participates in processes related to the immune system, including lymph-node development, lymphocyte differentiation, dendritic cell survival and T- cell activation, and tolerance induction [18]. Furthermore, osteoprotegerin, a natural decoy with similar effects to those of denosumab on RANK-L, may elicit beneficial effects to patients suffering from viral infections [19]. Therefore, denosumab may modulate the immune response associated to viral infections, such as SARS-CoV-2.

Non-inflammatory rheumatic conditions such as osteoporosis, osteoarthritis and fibromyalgia are all characterized by a high incidence of chronic pain and are mainly treated with classical non-steroidal anti-inflammatory drugs (NSAIDs) and opioids, but also with gabapentinoids and two particular antidepressant drugs, duloxetine and amitriptyline. In addition to chronic pain, these pathological conditions often present co-morbid emotional disorders that are treated with antidepressants. However, there is no data on the potential effects of these drugs on COVID-19 incidence.

Here, in order to elucidate the possible effects of anti-osteoporosis drugs (anti-resorptives, calcium and vitamin D) and associated treatments (analgesics and antidepressants) on COVID-19 incidence and clinical expression, we carried out a cross-sectional study of the cumulative incidence of COVID-19 in rheumatic patients suffering from non-inflammatory conditions and living in the influence area of a referral hospital in Barcelona, Spain.

## RESULTS

A total of 2,498 individuals were examined for eligibility and 2,102 fulfilled the inclusion criteria and were included in the analysis, 80.5% of which were women. Table 1 shows the description of the studied population and the distribution of COVID-19 across studied variables. The mean age was 66.4 years (SD, 13.3) and 63.7% of the population had osteoarthritis, 43.5% osteoporosis and 27.2% fibromyalgia. The most prevalent coexisting conditions were hypertension (42.4%), pulmonary disease (15.0%), cardiovascular (CV) disease (14.9%) and diabetes (12.6%). Regarding treatments, 62% were treated with vitamin D, 23.3% with calcium, 12.6% with denosumab, and 8.5% with intravenous zoledronate. More than a half of the population was exposed to analgesics and almost a third to antidepressants, mainly serotonin reuptake inhibitors (SSRIs) (15.8%) and dual-action antidepressants (13.2%).

**Table 1:** Characteristics of the study population and distribution of confirmed or hsCOVID-19 cases.

**Characteristic**	**All population (N=2102)**	**Confirmed or hsCOVID-19**	
		**No (N=1993)**	**Yes (N=109)**
Men	409 (19.5%)	388 (19.5%)	21 (19.3%)
Women	1693 (80.5%)	1605 (80.5%)	88 (80.7%)
Age [mean (SD)]	66.4 (13.3)	66.5 (13.3)	65.7 (13.2)
**Non-inflammatory rheumatic diagnosis^1^**			
Osteoarthritis	1340 (63.7%)	1263 (63.4%)	77 (70.6%)
Osteoporosis	914 (43.5%)	880 (44.2%)	34 (31.2%)
Fibromyalgia	571 (27.2%)	539 (27.0%)	32 (29.4%)
**Coexisting conditions**			
Diabetes	264 (12.6%)	245 (12.3%)	19 (17.4%)
Hypertension	892 (42.4%)	845 (42.4%)	47 (43.1%)
Pulmonary disease	315 (15.0%)	290 (14.6%)	25 (22.9%)
CV disease	314 (14.9%)	286 (14.4%)	28 (25.7%)
Cancer or active treatment	121 (5.76%)	115 (5.77%)	6 (5.50%)
Chronic kidney disease	114 (5.42%)	104 (5.22%)	10 (9.17%)
History of organ transplantation	9 (0.43%)	6 (0.30%)	3 (2.75%)
Any of these comorbidities	1232 (58.6%)	1159 (58.2%)	73 (67.0%)
**Treatments followed**			
Denosumab	264 (12.6%)	256 (12.8%)	8 (7.34%)
Intravenous Zoledronate	179 (8.52%)	173 (8.68%)	6 (5.50%)
Oral bisphosphonates	143 (6.80%)	136 (6.82%)	7 (6.42%)
Teriparatide	25 (1.19%)	25 (1.25%)	0 (0.00%)
Calcium	490 (23.3%)	474 (23.8%)	16 (14.7%)
Vitamin D	1303 (62.0%)	1241 (62.3%)	62 (56.9%)
Thiazide diuretics	262 (12.5%)	248 (12.4%)	14 (12.8%)
SERMs	11 (0.52%)	11 (0.55%)	0 (0.00%)
Analgesics	1220 (58.0%)	1154 (57.9%)	66 (60.6%)
Gabapentin	164 (7.80%)	153 (7.68%)	11 (10.1%)
Pregabalin	146 (6.95%)	134 (6.72%)	12 (11.0%)
Opioids	546 (26.0%)	510 (25.6%)	36 (33.0%)
Other Analgesics	959 (45.6%)	906 (45.5%)	53 (48.6%)
Antidepressants	657 (31.3%)	612 (30.7%)	45 (41.3%)
Tricyclic antidepressants	124 (5.90%)	116 (5.82%)	8 (7.34%)
Amitriptyline	102 (4.85%)	94 (4.72%)	8 (7.34%)
Others	22 (1.05%)	22 (1.10%)	0 (0.00%)
Dual-action antidepressants	277 (13.2%)	260 (13.0%)	17 (15.6%)
Duloxetine	207 (9.85%)	198 (9.93%)	9 (8.26%)
Venlafaxine	60 (2.85%)	53 (2.66%)	7 (6.42%)
Others	10 (0.48%)	9 (0.45%)	1 (0.92%)
SSRIs antidepressants	333 (15.8%)	307 (15.4%)	26 (23.9%)
Reboxetine	2 (0.10%)	2 (0.10%)	0 (0.00%)
Trazodone	33 (1.57%)	31 (1.56%)	2 (1.83%)
Glucocorticoids	60 (2.85%)	53 (2.66%)	7 (6.42%)
Inhaled Glucocorticoids	189 (8.99%)	172 (8.63%)	17 (15.6%)
Anti-hypertensive drugs	646 (30.7%)	610 (30.6%)	36 (33.0%)
ACE inhibitors	363 (17.3%)	344 (17.3%)	19 (17.4%)
ARBs	290 (13.8%)	273 (13.7%)	17 (15.6%)
Chronic NSAIDs	318 (15.1%)	301 (15.1%)	17 (15.6%)
Synthetic DMARDs	30 (1.43%)	26 (1.30%)	4 (3.67%)
Biologic DMARDs	1 (0.05%)	1 (0.05%)	0 (0.00%)
**COVID-19 status**			
Grade of hsCOVID-19 symptomatology			
Mild	63 (3.00%)	NA	63 (57.8%)
Moderate	16 (0.76%)	NA	16 (14.7%)
Severe	30 (1.43%)	NA	30 (27.5%)
COVID-19 Evolution:			
Home	71 (3.38%)	NA	71 (65.1%)
Hospitalization	25 (1.19%)	NA	25 (22.9%)
NIV	3 (0.14%)	NA	3 (2.75%)
ICU	1 (0.05%)	NA	1 (0.92%)
Death	9 (0.43%)	NA	9 (8.26%)
Positive SARS-CoV-2 test (PCR)	38 (1.81%)	NA	38 (34.9%)
Radiography:		NA	
Pathologic unilateral	13 (0.62%)	NA	13 (11.9%)
Pathologic bilateral	23 (1.09%)	NA	23 (21.1%)
COVID-19 diagnosed by PCR or radiography	48 (2.28%)	NA	48 (44.0%)

^1^Some individuals have more than one diagnosis.

ACE = angiotensin-converting enzyme. ARBs = angiotensin II receptor blockers. CV = cardiovascular disease. DMARDs = disease modifying anti-rheumatic drugs. hsCOVID-19 = highly suspected COVID-19 cases. ICU = intensive care unit. NA = not applicable. NIV = non-invasive ventilation. NSAIDs = non-steroid anti-inflammatory drugs. PCR = polymerase chain reaction. SERMs = selective estrogen receptor modulator. SD = standard deviation. SSRIs = selective serotonin reuptake inhibitors.

A total of 109 individuals had COVID-19 diagnosis (hereafter, COVID-19-positive or “COVID-19+” patients), representing 5.19% of the individuals included. They presented a higher prevalence of diabetes, CV disease, pulmonary disease and chronic kidney disease than those not diagnosed with COVID-19 (hereafter, COVID-19-negative “COVID-19-” patients). In terms of treatments, the exposure to denosumab, intravenous zoledronate, vitamin D, and selective estrogen receptor modulators (SERMs) was lower for COVID-19+ than for COVID-19- patients, whereas antidepressant treatment was more frequent in COVID-19-positive individuals.

As shown in Table 2, the age-standardized cumulative incidence rate in our population was 4.68% (CI95% 3.78-5.59%), being slightly higher than that in the general population of Barcelona (3.69%; CI95% 3.66-3.73%). However, when stratifying by the presence of osteoporosis, osteoarthritis and fibromyalgia, patients with osteoporosis presented lower rates (2.98%, CI95% 1.88-4.08) than the general population, whereas patients with osteoarthritis (4.58%, CI95% 3.46-5.70) and fibromyalgia (4.45%, CI95% 2.76-6.14) showed slightly higher rates.

**Table 2:** Crude and age-adjusted cumulative incidence rates of confirmed or hsCOVID-19 cases in our cohort and in the population of Barcelona (reference population) registered from March 1^st^ to May 3^rd^, 2020, stratified by the diagnosis of osteoporosis, osteoarthritis and fibromyalgia.

**Age group (years)**	**Incidence rate of confirmed or hsCOVID-19 cases in Barcelona**	**Confirmed or hsCOVID-19 cases in our cohort**							
		**All population**		**Population with osteoporosis**		**Population with osteoarthritis**		**Population with fibromyalgia**	
		**Crude cumulative incidence rate**	**Age-adjusted cumulative incidence rate**	**Crude cumulative incidence rate**	**Age-adjusted cumulative incidence rate**	**Crude cumulative incidence rate**	**Age-adjusted cumulative incidence rate**	**Crude cumulative incidence rate**	**Age-adjusted cumulative incidence rate**
20-29	4818 / 195194 (2.47%)	0 / 0 (0%)	0%	0 / 0 (0%)	0%	0 / 0 (0%)	0%	0 / 0 (0%)	0%
30-39	6628 / 250517 (2.65%)	2 / 44 (4.55%)	0.84%	0 / 5 (0%)	0%	0 / 10 (0%)	0%	2 / 31 (6.45%)	1.19%
40-49	7515 / 255707 (2.94%)	11 / 172 (6.40%)	1.20%	1 / 20 (5.00%)	0.94%	5 / 61 (8.20%)	1.54%	6 / 116 (5.17%)	0.97%
50-59	7807 / 218163 (3.58%)	28 / 409 (6.85%)	1.10%	6 / 110 (5.45%)	0.87%	18 / 220 (8.18%)	1.31%	15 / 208 (7.21%)	1.15%
60-69	5061 / 177078 (2.86%)	26 / 545 (4.77%)	0.62%	8 / 234 (3.42%)	0.44%	20 / 371 (5.39%)	0.70%	6 / 147 (4.08%)	0.53%
70-79	5147 / 143113 (3.60%)	23 / 545 (4.22%)	0.44%	8 / 295 (2.71%)	0.28%	19 / 391 (4.86%)	0.51%	3 / 52 (5.77%)	0.61%
80-89	8581 / 97289 (8.82%)	15 / 327 (4.59%)	0.33%	7 / 208 (3.37%)	0.24%	12 / 252 (4.76%)	0.34%	0 / 10 (0%)	0%
90 +	4795 / 25924 (18.5%)	4 / 47 (8.51%)	0.16%	4 / 38 (10.53%)	0.20%	3 / 32 (9.38%)	0.18%	0 / 1 (0%)	0%
All	3.69%	5.19%	4.68%	3.72%	2.98%	5.75%	4.58%	5.60%	4.45%
(CI95%)	(3.66-3.73%)	(4.24-6.13%)	(3.78-5.59%)	(2.49-4.95%)	(1.88-4.08%)	(4.5-6.99%)	(3.46-5.70%)	(1.71-9.50%)	(2.76-6.14%)

hsCOVID-19 = highly suspected COVID-19.

CI95% = 95% confidence intervals.

**Figure 1:**
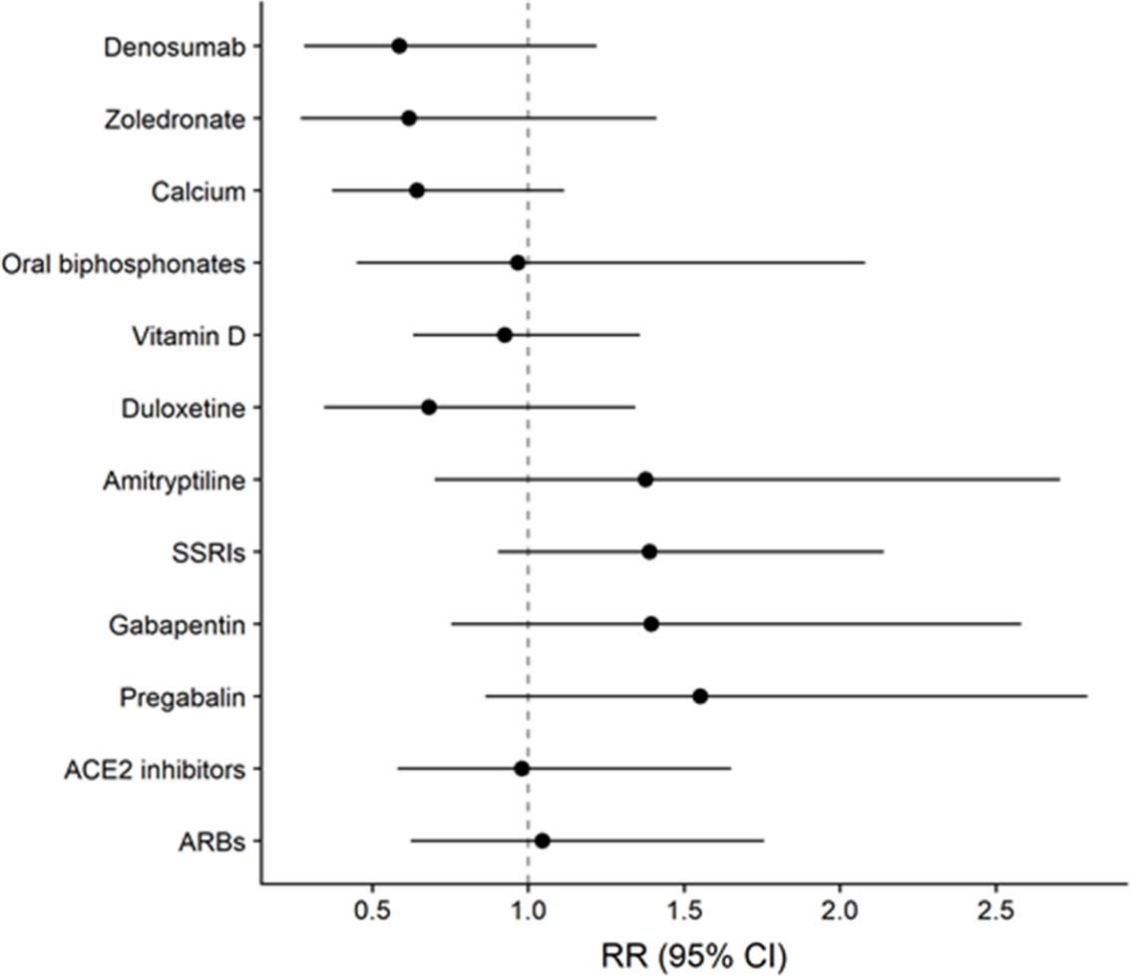
**Relative Risk (RR) with 95% Confidence Interval (CI95%) of COVID-19 diagnosis according to the exposure to different treatments, adjusted by sex, age, CV disease, diabetes, pulmonary disease, chronic kidney disease and active cancer or treatments.** The effect of Denosumab Zoledronate, Calcium, Oral bisphosphonates and Vitamin D were obtained from Model 1. Estimates for Duloxetine, SSRIs, Gabapentin, Pregabalin, ACE inhibitors and ARBs were obtained from Model 2.

Adjusted associations between different exposure variables (clinical characteristics and treatments) and COVID-19 diagnosis are shown in Table 3. CV disease was the comorbidity showing the highest RR of COVID-19 (RR=1.84; CI95%1.17-2.87), followed by chronic kidney disease, diabetes and pulmonary disease. Patients suffering from cancer or in active cancer treatment did not show an increased RR for COVID-19 diagnosis. Regarding treatments, the RR for COVID-19 was 0.58 (CI95%0.28-1.22) for denosumab, 0.62 (CI95%0.27-1.41) for intravenous zoledronate and 0.64 (CI95%0.37-1.12) for calcium. No association between COVID-19 and oral bisphosphonates, vitamin D or thiazide diuretics was found. Analgesics, particularly pregabalin (RR=1.55; CI95%0.86-2.79), gabapentin (RR=1.39; CI95%0.75-2.58) and opioids (RR=1.25; CI95%0.85-1.83) showed an increased RR for COVID-19. In the case of antidepressants, SSRIs presented an RR of 1.54 (CI95%1.00-2.36). The tricyclic antidepressant amitriptyline presented an RR of 1.38 (CI95% 0.7, 2.71) and the RR of all dual-action antidepressants together was 1.22 (CI95% 0.72, 2.08). In sharp contrast, the RR of the dual-action antidepressant duloxetine was 0.68 (CI95% 0.34-1.34). Figure 1 summarizes the adjusted RR for the incidence of COVID-19 according to the exposure to the most prevalent studied treatments.

**Table 3:** Adjusted Relative Risk (aRR) with 95% confidence intervals (CI95%) of confirmed or hsCOVID-19 according to the presence of several comorbidities and treatments.

	**N (All = 2102)**	**Model 1 - aRR (CI95%)**	**Model 2 - aRR (CI95%)**
Women	1693	1.12 (0.71, 1.76)	1.11 (0.7, 1.76)
Age		0.99 (0.98, 1.01)	0.99 (0.98, 1.01)
**Comorbidities**			
CV disease	314	1.84 (1.17, 2.87)	1.86 (1.19, 2.91)
Diabetes	264	1.2 (0.71, 2.03)	1.19 (0.7, 2.03)
Pulmonary disease	315	1.36 (0.76, 2.45)	1.34 (0.73, 2.46)
Chronic kidney disease	114	1.58 (0.82, 3.07)	1.56 (0.8, 3.03)
Cancer or active treatment	121	1.06 (0.46, 2.46)	1.06 (0.45, 2.47)
**Treatments followed**			
Denosumab	264	0.58 (0.28, 1.22)	0.59 (0.28, 1.23)
Intravenous Zoledronate	179	0.62 (0.27, 1.41)	0.61 (0.27, 1.38)
Oral bisphosphonates	143	0.97 (0.45, 2.08)	0.97 (0.46, 2.06)
Calcium	490	0.64 (0.37, 1.12)	0.64 (0.37, 1.11)
Vitamin D	1303	0.92 (0.63, 1.36)	0.91 (0.62, 1.34)
Thiazide diuretics	262	0.95 (0.54, 1.67)	0.94 (0.53, 1.66)
Analgesics	1220	0.92 (0.61, 1.38)	
Gabapentin	164		1.39 (0.75, 2.58)
Pregabalin	146		1.55 (0.86, 2.79)
Opioids	546		1.25 (0.85, 1.83)
Other Analgesics	959		0.94 (0.64, 1.37)
Dual-action antidepressants	277	1.22 (0.72, 2.08)	
Duloxetine	207		0.68 (0.34, 1.34)
Tricyclic antidepressants	124	1.06 (0.54, 2.08)	
Amitriptyline	102		1.38 (0.7, 2.71)
SSRIs antidepressants	333	1.54 (1, 2.36)	1.39 (0.9, 2.14)
Inhaled Glucocorticoids	189	1.42 (0.73, 2.77)	1.39 (0.7, 2.74)
Anti-hypertensive drugs	646	1.06 (0.7, 1.6)	
ACE inhibitors	363		0.98 (0.58, 1.65)
ARBs	290		1.05 (0.62, 1.76)
Chronic NSAIDs	318	0.94 (0.57, 1.56)	0.95 (0.58, 1.55)

ACE = angiotensin-converting enzyme. ARBs = angiotensin II receptor blockers. CV = cardiovascular disease. hsCOVID-19 = highly suspected COVID-19 cases. NSAIDs = non-steroid anti-inflammatory drugs. SSRIs = selective serotonin reuptake inhibitors.

Finally, the RR estimates using propensity score matching for the exposure to denosumab, intravenous zoledronate and calcium are included in Supplementary Table 3. The resulting RR estimates were almost equivalent to the unmatched database, with values of 0.73 (CI95% 0.30- 1.78) for denosumab, 0.55 (CI95% 0.20-1.44) for intravenous zoledronate and 0.72 (CI95% 0.39-1.37) for calcium. The adjusted RRs by the other treatments did not differ from the crude ones: 0.87 (CI95% 0.30-2.52) for denosumab, 0.43 (CI95% 0.37-1.35) for intravenous zoledronate and 0.67 (CI 95% 0.36-1.27) for calcium.

## DISCUSSION

The present study reveals that the main treatments currently used for osteoporosis are not associated with an increase in COVID-19 incidence. All the treatments analyzed in our study were continued after the presentation of COVID-19 symptoms following the recommendations of multiple international rheumatology [13, 14] and bone field organizations [15, 16]. Interestingly, the exposure to two anti-resorptive drugs, denosumab (RR=0.58; CI95% 0.28, 1.22) and intravenous zoledronate (RR=0.62; CI95% 0.27, 1.41), was associated with a 40% decreased risk of COVID-19. Recent relevant studies underline the high predictive value of the RR points estimate in these Poisson regression models, which often present high confidence interval ranges [20, 21].

The anti-osteoporosis target of denosumab is the RANK/RANKL system involved in the inhibition of osteoclastogenesis. The RANK/RANK-L system also participates in immune responses, including lymph-node development, lymphocyte differentiation, dendritic cell survival and T-cell activation [18]. Denosumab prevents RANK-L from binding to RANK receptor, thereby inhibiting osteoclast differentiation. RANK-L inhibition by denosumab modifies immune cell profiles and decreases the activity of pro-inflammatory cytokines [22]. This decrease in the inflammatory responses might elicit beneficial effects during viral infections, as previously reported with other RANK-L inhibitors [19], and could explain the decreased incidence of COVID-19 cases among patients treated with denosumab. Indeed, COVID-19 progression has been associated with a hyperactivity of pro-inflammatory cytokines [5] and a dysregulation of the immune system related to an abnormal monocytes/macrophage/dendritic cells response [6], which could be attenuated by denosumab treatment.

In contrast to denosumab, bisphosphonates bind to hydroxyapatite crystals and inhibit mature osteoclast function through induction of apoptotic pathways or blockade of cytoskeletal assembly by inhibition of lipid modification of associated proteins [23]. The exposure to intravenous zoledronate, but not to other oral bisphosphonates, showed a negative association with the incidence of COVID-19. The higher potency of intravenous zoledronate in comparison to any other oral bisphosphonate used in this study [24] may explain the differential result obtained with both groups of bisphosphonates. Intravenous zoledronate treatment reduces mortality after hip fracture [25], which may be related to decreased risk of CV disease, general health status improvement, fracture prevention, improved regulation of the immune system and a reduced incidence of pneumonia [26, 27]. Interestingly, zoledronate may make dendritic cells and their precursors less susceptible to SARS-CoV-2 infection, which could explain the beneficial effects here reported on COVID-19 incidence [28]. Indeed, zoledronate inhibits the prenylation of small GTPases [29, 30], which may hinder endosomal exocytosis in the dendritic cells required for the advance of SARS-CoV-2 infection. These protective effects on dendritic cells and their precursors may lead to immune-stimulation of T cell expansion and enhanced activity of natural killer cells, crucial mechanisms to prevent the progression of SARS-CoV-2 infection in the lung [31].

In the present study, patients treated with calcium supplements also presented a decreased risk of COVID-19 (RR=0.64, CI95% 0.37, 1.12). In agreement, recent studies have reported reduced COVID-19 mortality in patients treated with calcium [32]. A decrease in total and ionized calcium blood levels has been reported in COVID-19 patients [33]. Changes in calcium levels in COVID-19 patients may be due to alterations in intestinal absorption, imbalance in regulatory mechanism involving parathyroid hormone and vitamin D, or to a direct effect caused by SARS- CoV-2 [33]. However, our results are compatible with no relevant effect of vitamin D on COVID-19 incidence (RR=0.97, CI95% 0.45, 2.08). A possible mechanism that may explain the beneficial effects of calcium in COVID-19 found in our study could be related to the action of calcium, through a specific calcium-based signal, on the generation of two immune cell types: T follicular helper cells and T follicular regulatory cells. These T cells promote an appropriate immune response against infectious agents, such as viruses [33, 34]. Accordingly, calcium supplements may counteract the decreased serum levels of calcium promoted by SARS-CoV-2 infection, which may lead to an improvement of the immune cell response and attenuate the probability of infection progression.

In agreement with the protective effects shown by the exposure to the main anti-osteoporosis treatments, age-adjusted cumulative incidence of COVID-19 in osteoporosis patients was lower (RR=2.98%, CI95% 1.88, 4.08) than in all the patients included in this study (RR=4.68%, CI95% 3.78-5.59%). This decrease was not observed with other non-inflammatory rheumatic conditions analyzed in our study. The comorbidity that produced the highest positive association with COVID-19 incidence in non-inflammatory rheumatic conditions was CV disease (RR=1.84, CI95% 1.17, 2.87;), although other co-morbidities, such as chronic kidney disease (RR=1.58, CI95% 0.82, 3.07) also substantially enhanced this incidence, as expected.

In our study, the exposure to different antidepressant drugs produced various effects on COVID-19 incidence. Interestingly, the dual acting serotonin/norepinephrine inhibitor duloxetine decreased the incidence of COVID-19 (RR=0.68, CI95% 0.34, 1.34). Antidepressant drugs have been postulated to modulate immune responses by modifying the serotonin/norepinephrine equilibrium, which modifies the balance of the different T cell populations involved in the release of cytokines [35]. Antidepressants that change this serotonin/norepinephrine balance, such as the dual inhibitor duloxetine, may facilitate the maintenance or restauration of an appropriate T cells equilibrium and cytokine production [35]. However, amitriptyline, a tricyclic antidepressant that also has this dual serotonin/norepinephrine inhibitory effect, showed a different profile than duloxetine on the incidence of COVID-19. Both antidepressants have a completely different activity on other receptors [36], including antagonist activity of duloxetine on sigma-1 receptors [37], a mechanism postulated as a target of interest for re-purposing compounds for COVID-19 treatment [38]. Interestingly, SSRIs cause bone loss by a mechanism that counteracts local anti-resorption [39], in contrast to anti-osteoporosis medications that decrease COVID-19 incidence in our study. Also, opposite to anti-osteoporosis medications, patients taking SSRIs presented a 50% enhanced risk of COVID-19 (RR=1.54, CI95% 1.0, 2.36).

Our results suggest a positive association between the exposure to gabapentinoids and COVID-19 incidence. This increased risk was mainly revealed after pregabalin exposure (RR=1.55, CI95% 0.86, 2.79), an anti-epileptic drug mainly used in our population for chronic pain treatment. Pregabalin predominantly blocks the alpha2-delta subunit of voltage-gated calcium channels [40] and decreases immune responses under chronic pain conditions [41]. However, chronic pregabalin administration in HIV patients increased T cell levels in blood suggesting a possible activation of the immune response under this particular condition [42]. Moreover, SARS-CoV-2 binds to angiotensin-converting-enzyme-2 (ACE2) receptors and pregabalin has been reported to decrease these receptors in animal models [43]. When the amount of ACE2 is reduced due to the virus occupancy, individuals could be more susceptible to severe COVID-19 illness because enough ACE2 is still available for viral entry, whereas this decreased ACE2 availability facilitates angiotensin II-mediated injury. This pathophysiological mechanism associated to reduced ACE2 expression may promote inflammation, cell death and organ failure, mainly in the heart and lungs [44]. This mechanism may also contribute to the increased risk of COVID-19 we observed here among patients treated with pregabalin.

Our results from the two groups of anti-hypertensive drugs analyzed, ACE2 inhibitors and angiotensin II receptor blockers (ARBs), are compatible with no effect on the incidence of COVID-19. These results are in agreement with a recent meta-analysis showing that the use of these anti-hypertensive drugs in patients with COVID-19 does not increase the risk of SARS-CoV-2 infection and COVID-19 severity, being these treatments associated with a decreased risk of mortality [45]. Therefore, all these results suggest that treatment with ACE2 and ARBs should be continued in COVID-19 patients who are taking these anti-hypertensive medications.

Some limitations of this study must be addressed. Osteoporosis grade and related comorbidities may have biased the risk estimates in our study. To control for the potential effect of confounding by indication, RRs of COVID-19 were also estimated after propensity score matching with the main available covariates that predict receiving denosumab, zoledronate or calcium treatment and the RRs obtained were also negatively associated with COVID-19. The similar results obtained with denosumab, zoledronate and calcium using both analyses suggest that the effects of these anti-osteoporosis medications on COVID-19 were not due to the presence of osteoporosis or underlying comorbidities. Furthermore, the data in this cross-sectional study were collected from a large number of patients. Thus, data have been collected by different researchers and some data may have been missed or slight differences in classification criteria may have been applied. Notwithstanding, all of the researchers were expert clinicians or medical researchers, and several meetings were held among them to unify classification criteria and to review clinical records. Also due to the cross-sectional design of the study, the degree of severity of some patients may have changed throughout the survey and these changes may not have been assessed. Additionally, our study cohort included patients from a tertiary hospital that were probably suffering from more severe forms of non-inflammatory rheumatic conditions in comparison with patients in primary or secondary care settings, which may introduce bias. However, all the anti-osteoporosis medications were uniformly covered by the public health insurance system in Spain, thus avoiding biases related medication costs. Finally, some asymptomatic patients may not have been registered due to the low availability of tests for SARS-Cov-2 in our country in the early stages of the pandemic.

In summary, our results reveal that chronic treatment with some of the main anti-osteoporosis drugs currently available, anti-resorptives, calcium and vitamin D, are not associated with increased risk of COVID-19. In contrast, a decreased incidence of COVID-19 was revealed with two anti-resorptives drugs, denosumab and zoledronate, as well as with calcium treatment. Some of the pain treatments used in these non-inflammatory rheumatic conditions may influence COVID-19 outcomes, since the incidence of COVID-19 was decreased in patients treated with duloxetine and increased in those taking pregabalin. In conclusion, our data are consistent with a lack of direct relationship between osteoporosis therapies and COVID-19 incidence, providing scientific evidence in support of the recently-published guidelines by the ACR, EULAR, ASBMR and IOF [13–16] to maintain anti-osteoporosis treatments for COVID-19 patients, which were based solely on expert opinions.

## MATERIALS AND METHODS

### Study design, and population

A cross-sectional study was performed at the Rheumatology Service of Hospital del Mar (Barcelona, Spain) that includes patients diagnosed with osteoporosis, osteoarthritis and/or fibromyalgia. Patients receiving care at the outpatient Rheumatology Service for the last six months were eligible. The exclusion criteria were <18 years old, previous death not related with SARS- CoV-2 infection, presence of immune-mediated inflammatory disease, a negative SARS-CoV-2 test, or failing to follow up at the primary care center during the studied period.

### Outcomes

Hospital and primary care clinical history revision have been performed and the patient data included in this study were collected from March 1^st^ to May3^rd^, 2020, the period of the highest COVID-19 incidence in Spain. The primary outcome was the presence of COVID-19 diagnosis, although other related variables were also recorded, including PCR results, lung radiography, symptomatology and evolution. At the time of revision, demographic and clinical data were also collected, with a particular focus on comorbidities (Supplementary Table 1) and medical drug prescriptions (Supplementary Table 2).

### Statistical analysis

Cumulative incidence was adjusted for age by direct standardization using a COVID-19 epidemiological database (RSAcovid19) generated by the Department of Health of the Government of Catalonia [39]. This database contains daily cumulative positive cases and daily cumulative suspicious cases activated by the epidemiological surveillance service. For this analysis, we selected positive or suspicious cases registered from March 1^st^ to May 3^rd^, 2020 in the city of Barcelona, which was the reference population for direct standardization [40]. The RSAcovid19 database considers as positive the cases that tested positive in a diagnostic test (PCR, rapid test, or ELISA test), and those confirmed by an epidemiologist as a positive case while they consider as “suspicious cases” those who had symptoms classified by a health professional as a possible case, but without a diagnostic test.

To evaluate the associations between different treatments (with >100 exposed patients; reference category: unexposed) and the presence of COVID-19, Poisson regression models with robust variance estimation were used to estimate RR and 95% confidence intervals (CI95%). Models were adjusted by sex, age, diabetes, pulmonary disease, cardiovascular disease, chronic kidney disease, and active cancer or treatment. Model 1 included the following treatments: denosumab, oral/intravenous bisphosphonates, calcium, vitamin D, thiazide diuretics, analgesics, antidepressants (dual action vs tricyclic vs SSRIs), inhaled glucocorticoids, anti- hypertensive drugs and NSAIDs. Model 2 included the specific effect of the analgesics gabapentin, pregabalin, opioids and others; the dual-action antidepressant duloxetine; the tricyclic antidepressant amitriptyline; and two types of anti-hypertensive drugs: ACE2 inhibitors and ARBs; together with denosumab, oral/intravenous bisphosphonates, calcium, vitamin D, thiazide diuretics, inhaled glucocorticoids and chronic NSAIDs.

Finally, the presence of osteoporosis, together with the studied comorbidities (sex, age, cardiovascular disease, diabetes, pulmonary disease, kidney disease and cancer) were used to calculate the probability of treatment assignment for denosumab, bisphosphonates and calcium with propensity score matching based on the nearest neighbor method [41]. Therefore, each treated individual was matched with an untreated individual whose propensity score was closest to that of the treated subject. Statistical analyses were performed using R (R Foundation for Statistical Computing, Vienna, Austria) version 3.5.2.

### Ethics statement

All research in this study was conducted in accordance with the ethical standards of the Declaration of Helsinki and according to national and international guidelines. The observational study was approved by the Parc de Salut Mar Ethical Committee on Clinical Studies (ref. 2020/9246) before it started, and was monitored by the Clinical Trial Unit of Rheumatology Service at Hospital del Mar. Due to the nature of the study (all the data are completely anonymous), the importance of expedited results, and their implication for treatment of patients during the SARS-COV-2 pandemic, we did not obtain informed consent from the participants.

## Supplementary Material

Supplementary Tables
