# Supplementary material for: Influence of anti-osteoporosis treatments on the incidence of COVID-19 in patients with non-inflammatory rheumatic conditions
Source: Aging (Albany NY). 2020 Oct 20;12(20):19923–37. doi: 10.18632/aging.104117 (PMC7655189; doi:10.18632/aging.104117)
Supplement: Supplementary Tables [file aging-12-104117-s001..pdf]

SUPPLEMENTARY TABLES

Supplementary Table 1. Definition of the comorbidities registered.

Comorbidities	Definition
AHT	Diagnosis of arterial hypertension in actual treatment with antihypertensive drugs.
Diabetes	Diagnosis of diabetes, treated with diet, oral drugs or insulin administration.
Pulmonary disease	Any disease affecting respiratory system functionality and treated with any of the related drugs. Patients suffering from asthma, Chronic obstructive pulmonary disease (COPD), Pulmonary fibrosis, Obstructive sleep apnea syndrome (OSAS) and interstitial lung disease were specifically registered.
CV disease	Diagnosis of cardiovascular disease
Chronic kidney disease	Chronic kidney disease diagnosis, in any of the stages.
Active cancer or treatment	Patients in a cancer process or in treatment for a previous cancer, along the period studied (1st to the 29th of March)
Tissue or organ transplantation	Any transplantation throughout the patient's life, in actual treatment.

Supplementary Table 2. List of types of treatments registered and drugs belonging to each type.

Type of treatment	Drugs included	
Analgesics	NSAIDs	
	Opioids	
	Pregabalin	
	Gabapentin	
	Other analgesics	
Antidepressants	Dual Acting	Duloxetine
		Venlafaxine
		Others
	Tricyclic	Amitriptyline
		Other
	Selective serotonin reuptake inhibitors (SSRIs)	
	Reboxetine	
	Other antidepressants	
Antiresorptive Agents	Bisphosphonates	Oral
		Intravenous
	Denosumab	
	Selective estrogen receptor modulator (SERM)	
Anabolic Agents	Teriparatide	
Supplements for osteoporosis	Calcium	
	Vitamin D (in any of its forms or metabolits)	
	Thiazides diuretics	
Corticoids	Any type of oral corticoid (all converted to prednisone dose)	
	Any type of inhaled corticoids	
Antihypertensive drugs	Angiotensin-converting enzyme (Enalapril, Ramipril, Lisinopril, …)	
	Angiotensin II Receptor Blockers (Losartan, Valsartan, Candesartan…)	

Supplementary Table 3. Relative risk (RR) and 95% confidence interval (95% CI) of hsCOVID-19 after propensity score matching.

	Denosumab			Intravenous Zoledronate			Calcium		
**Sample size**		control	treated		control	treated		control	treated
	N All	1838	264	N All	1923	179	N All	1612	490
	N matched	264	264	N matched	179	179	N matched	490	490
	N unmatched	1574	0	N unmatched	1744	0	N unmatched	1122	0
**COVID-19 (matched data)**	Yes	11	8	Yes	11	6	Yes	22	16
	No	253	256	No	168	173	No	468	474
**Crude RR (95%CI)**	0.73 (0.30-1.78)			0.55 (0.20-1.44)			0.72 (0.39-1.37)		
**Adjusted RR (95%CI)***	0.87 (0.30-2.52)			0.43 (0.14-1.35)			0.67 (0.36-1.27)		
**Summary of balance for matched data**	Means Treated	Means Control	Mean Diff	Means Treated	Means Control	Mean Diff	Means Treated	Means Control	Mean Diff
Osteoporosis	0.97	0.97	0.00	0.92	0.92	0.00	0.77	0.77	0.00
Women	0.86	0.86	-0.01	0.77	0.80	-0.03	0.85	0.88	-0.02
Age	74.05	73.29	0.76	71.61	71.69	-0.08	71.34	71.57	-0.22
CV disease	0.17	0.13	0.04	0.18	0.17	0.02	0.17	0.16	0.01
Diabetes	0.12	0.10	0.02	0.18	0.17	0.01	0.10	0.12	-0.02
Pulmonary disease	0.20	0.21	-0.01	0.19	0.17	0.02	0.16	0.14	0.01
Renal disease	0.11	0.09	0.01	0.08	0.04	0.03	0.07	0.06	0.01
Cancer	0.09	0.08	0.02	0.15	0.15	0.01	0.08	0.08	0.00

CV= cardiovascular.

*RR for denosumab is adjusted by the exposure to calcium, bisphosphonates, vitamin D, thiazide, analgesics, dual antidepressants, tricyclic antidepressants, SSRI antidepressants (selective serotonin reuptake inhibitors), glucocorticoids, anti-hypertensive drugs and NSAIDs. RR for intravenous zoledronate is adjusted by the exposure to denosumab, oral bisphosphonates, calcium, vitamin D, thiazide, analgesics, dual antidepressants, tricyclic antidepressants, SSRI antidepressants, glucocorticoids, anti-hypertensive drugs and NSAIDs. RR for calcium is adjusted by the exposure to denosumab, bisphosphonates, vitamin D, thiazide, analgesics, dual antidepressants, tricyclic antidepressants, SSRI antidepressants, glucocorticoids, anti-hypertensive drugs and NSAIDs.
